# Bladder Explosion during Transurethral Resection of the Prostate with Nitrous Oxide Inhalation

**DOI:** 10.1155/2015/464562

**Published:** 2015-07-30

**Authors:** Eiko Hirai, Joho Tokumine, Alan Kawarai Lefor, Shinobu Ogura, Miwako Kawamata

**Affiliations:** ^1^Department of Anesthesia, Seikei-kai Chiba Medical Center, 1-11-12 Minami-cho, Chuo-ku, Chiba-shi, Chiba 260-0842, Japan; ^2^Department of Anesthesiology, Kyorin University School of Medicine, 6-20-2 Shinkawa, Mitaka-shi, Tokyo 181-8611, Japan; ^3^Department of Surgery, Jichi Medical University, 3311-1 Yakushiji, Shimotsuke-shi, Tochigi 329-0498, Japan; ^4^Department of Anesthesiology, Hakujikai Memorial General Hospital, 5-11-1 Shikahama, Adachi-ku, Tokyo 123-0864, Japan; ^5^Nippori Clinic, Medical Center East, Tokyo Women's Medical University, Station Port Tower 4th F 2-20-1 Nishinippori, Arakawa-ku, Tokyo 116-0013, Japan

## Abstract

Bladder explosions are a rare complication of transurethral resection of the prostate. We report a patient who suffered a bladder rupture following transurethral resection of the prostate. Although explosive gases accumulate during the procedure, a high concentration of oxygen is needed to support an explosion. This rare phenomenon can be prevented by preventing the flow of room air into the bladder during the procedure to maintain a low concentration of oxygen inside the bladder.

## 1. Introduction

An intravesical explosion can occur while using an electrical device such as a resectoscope during transurethral resection of prostate (TURP) [1, 2]. The resectoscope uses electrical energy to remove tissue and cauterize the remaining bladder. Tissue is evaporated by diathermy, which uses high-frequency electrical current and generates a great deal of heat. During TURP, small bubbles on an arc of the resectoscope are usually seen, which include hydrogen, oxygen, and other gases derived from the combustion of tissue and water [3–6]. Some of these gases, especially the hydrogen, are explosive [4, 5]. Occasionally, a “pop” sound can be heard during TURP, which suggests a subclinical intravesical explosion [1]. If the resectoscope causes a sufficient explosion, expansion of the intravesical gas results in increased internal pressure in the bladder which can tear the bladder wall [3–9]. We report a patient who suffered a bladder explosion at the end of a TURP, as well as a review of the literature and summary of the mechanism of explosion, and discuss how to prevent this complication.

## 2. Case Presentation

A 64-year-old man, ASA physical status class II, was scheduled to undergo TURP for benign prostatic hyperplasia and bladder stones with urinary obstruction.

When the patient entered the operating room, the blood pressure (BP) was 147/95 mmHg, heart rate (HR) was 59 min^−1^, and hemoglobin saturation (SpO_2_) was 100%. Spinal anesthesia was administered at the L3-L4 interspinous space, using 3 mL of 0.5% isobaric bupivacaine. The BP gradually decreased to 118/68 mmHg, the pulse was 65 min^−1^, and SpO_2_ was 98%. The level of spinal block was assessed with loss of cold sensation and determined to be below the thoracic 11 dermatome level. The patient looked anxious and was somewhat restless. We administrated a sedative using 2 mg of midazolam to induce calm.

The bladder was irrigated with UromaticS (Baxter, Japan) which contains 90 g of D-sorbitol in 3 liters (pH 4.5–6.5, 165 mOsm/L). During the operation, the patient described feeling dull pain. Nitrous oxide 1.5·L·min^−1^ and oxygen 1.5·L·min^−1^ were given by face mask. The pain was alleviated and the operation was performed. The operating time from start to resection of 6 g of the prostate and removal of two bladder stones was 85 mins.

When the resectoscope was removed, a loud “pop” sound was heard at the same time. The BP and the HR were transiently elevated to 147/83 mmHg and 87 min^−1^, respectively. The surgeon started bladder irrigation which returned bloody fluid. The BP decreased to 96/62 mmHg, and the HR was 65 min^−1^. The patient then described severe abdominal pain. Exploratory laparotomy was emergently performed. General anesthesia was induced with propofol and rocuronium and maintained with sevoflurane along with mixed air and oxygen. At laparotomy, the bladder was found to be ruptured with a large laceration. Other abdominal organs were not injured. The bladder was primarily repaired, and the operation was completed. During the open procedure, the BP and HR were 138–100/85–64 mmHg and 87–62/min, respectively. The SpO_2_ was maintained at 98–100% from the beginning of the operation till the procedure was completed.

The postoperative course was uneventful. The patient was discharged without symptoms of urinary dysfunction.

## 3. Discussion

Bladder explosion is a rare and dreadful complication during TURP. It was first reported in 1926 [10] and there are now 25 patients described in 19 reports [3, 5, 7, 8, 10–24]. Bladder rupture is estimated to occur in approximately 0.02% of transurethral procedures [25]. The degree of bladder injury secondary to an explosion varies from a loud “pop” sound only to a ruptured bladder needing surgical repair [1].

A likely mechanism for the intravesical explosion has been previously described [3, 4, 6, 8, 9]. Gases containing hydrogen are produced by electrolysis of intracellular water. These gases accumulate in the bladder, which contains about 5% oxygen during the procedure. Air from the room can enter the bladder while changing the irrigation bag, or inappropriate handling of the evacuator bulb. Room air is 21% oxygen, which is sufficient to support an explosion. The heated resectoscope in the presence of explosive hydrogen gas with a sufficient amount of oxygen then triggers an explosion.

A variety of conditions in patients suffering intravesical explosions have been reported. In 10 previously reported cases, tumors were present at the bladder dome [4, 7, 12, 15–21]. The occurrence of an explosion may be related to the presence of a gas layer with trapped air at the dome of the bladder. High power electrocautery was used for coagulation in situations with difficult hemostasis (three patients) [7, 10, 24]. In other reported cases, the explosion occurred after evacuating resected tissues (five patients) or changing the irrigation bag (one patient) [3–5, 12, 14]. In each of these cases, room air was noted to have entered the bladder. Bladder rupture was noted at the end of the operation in 11 patients [5, 8, 11, 13, 17, 21, 23]. The explosion may have been related to gas accumulated in the bladder at the end of the procedure.

In previously reported cases, various types of anesthesia have been used. General anesthesia was used in three cases, including two with inhalation agents and one with intravenous anesthesia [5, 11, 12]. Caudal epidural anesthesia was used in two cases [11]. Spinal anesthesia was used in seven cases [18, 19, 22–24]. In the remaining 13 reported cases, the type of anesthesia was not reported. In the present patient, the diagnosis of a major intra-abdominal injury may have been facilitated by the patient's ability to report the sudden onset of severe abdominal pain.

Nitrous oxide was used in one previously reported case [11], in which no explosion occurred. Therefore, the present patient may be the first report of a bladder explosion using nitrous oxide. The instruction manuals of the resectoscope and some investigators have warned not to use nitrous oxide while using the electrocautery [24]. Nitrous oxide may increase the risk of bladder explosion because of its character of combustibility and volume expansion in closed spaces.

The resectoscope is routinely used during TURP. Explosive gases are always present inside the bladder. A prevention strategy should be considered to avoid an increased risk of this serious complication. Furthermore, all members for the surgical team should know their roles to keep the patient safe. Surgeons should limit the power used during coagulation and cutting to the minimum needed for effectiveness, which will lead to accumulation of a smaller volume of explosive gases in the bladder [3–5, 7, 8]. Surgeons should prevent room air from entering the bladder while handling the evacuator bulb [1, 3, 7, 14, 26] and nurses can help to prevent the entry of air into the system while changing the irrigation bag [1, 5]. Background music should be stopped during the operation so that the characteristic “pop” sound could more easily be heard if it occurs [2]. In patients with tumors located at the 12 o'clock position, the anesthesiologist may discuss with the urologist to change the bed angle to avoid accumulation of explosive gases at the bladder dome [2, 15]. The anesthesiologist must be a good communicator in the operating room with all members of the operating room team.
